# Microglia amplify inflammatory activation of astrocytes in manganese neurotoxicity

**DOI:** 10.1186/s12974-017-0871-0

**Published:** 2017-05-05

**Authors:** Kelly S. Kirkley, Katriana A. Popichak, Maryam F. Afzali, Marie E. Legare, Ronald B. Tjalkens

**Affiliations:** 1 Department of Environmental and Radiological Health Sciences, College of Veterinary Medicine and Biomedical Sciences, Colorado State University, 1680 Campus Delivery, Fort Collins, CO 80523-1680 USA; 2 Center for Environmental Medicine, Colorado State University, Fort Collins, CO USA; 3 Program in Molecular, Cellular and Integrative Neuroscience, Colorado State University, Fort Collins, CO USA

**Keywords:** Neuroinflammation, Glial crosstalk, Manganism, NF-κB, Tumor necrosis factor

## Abstract

**Background:**

As the primary immune response cell in the central nervous system, microglia constantly monitor the microenvironment and respond rapidly to stress, infection, and injury, making them important modulators of neuroinflammatory responses. In diseases such as Parkinson’s disease, Alzheimer’s disease, multiple sclerosis, and human immunodeficiency virus-induced dementia, activation of microglia precedes astrogliosis and overt neuronal loss. Although microgliosis is implicated in manganese (Mn) neurotoxicity, the role of microglia and glial crosstalk in Mn-induced neurodegeneration is poorly understood.

**Methods:**

Experiments utilized immunopurified murine microglia and astrocytes using column-free magnetic separation. The effect of Mn on microglia was investigated using gene expression analysis, Mn uptake measurements, protein production, and changes in morphology. Additionally, gene expression analysis was used to determine the effect Mn-treated microglia had on inflammatory responses in Mn-exposed astrocytes.

**Results:**

Immunofluorescence and flow cytometric analysis of immunopurified microglia and astrocytes indicated cultures were 97 and 90% pure, respectively. Mn treatment in microglia resulted in a dose-dependent increase in pro-inflammatory gene expression, transition to a mixed M1/M2 phenotype, and a de-ramified morphology. Conditioned media from Mn-exposed microglia (MCM) dramatically enhanced expression of mRNA for *Tnf*, *Il-1β*, *Il-6*, *Ccl2*, and *Ccl5* in astrocytes, as did exposure to Mn in the presence of co-cultured microglia. MCM had increased levels of cytokines and chemokines including IL-6, TNF, CCL2, and CCL5. Pharmacological inhibition of NF-κB in microglia using Bay 11-7082 completely blocked microglial-induced astrocyte activation, whereas siRNA knockdown of *Tnf* in primary microglia only partially inhibited neuroinflammatory responses in astrocytes.

**Conclusions:**

These results provide evidence that NF-κB signaling in microglia plays an essential role in inflammatory responses in Mn toxicity by regulating cytokines and chemokines that amplify the activation of astrocytes.

## Background

Manganism or manganese neurotoxicity is a disease caused by chronic exposure to elevated levels of the essential trace metal manganese (Mn) and is marked by clinical motor deficits caused by neuropathology within the basal ganglia of affected individuals [1–3]. The pathogenic events that lead to neuronal loss in this disease are largely undefined. A number of studies in rodent and non-human primates treated with Mn have attributed the progression of injury to the presence of neuroinflammation and increased expression of pro-inflammatory factors such as tumor necrosis factor-alpha (TNF), interleukin 1-beta (IL-1β), and nitric oxide synthase 2 (NOS2) [4, 5]. Activated glia are implicated as the primary source of these pro-inflammatory factors, given that Mn potentiates inflammatory gene expression in astrocytes [6–9] and the presence of glia can significantly lower the dose of Mn required to induce neuronal loss [10]. However, the mechanisms underlying these neuroinflammatory effects and the role of microglia have long been poorly understood.

Microglia are the primary immune effector cells of the central nervous system and play an important role in the response of the brain to both foreign and endogenous insults [11]. When activated, microglia transition from a resting, ramified cellular phenotype to a rod-like morphology and finally to a phagocytic, amoeboid phenotype associated with expression of neuroinflammatory genes [12, 13]. As a myeloid-derived cell, activated microglia can polarize into two distinct macrophage subtypes, M1 or M2. The polarization of microglia is facilitated by the microenvironment at sites of injury and ultimately dictates the effect of the neuroinflammatory response [14, 15]. The M1 phenotype is associated with prototypic inflammatory responses with increased release of inflammatory cytokines and oxidative/nitrative compounds including TNF, interleukins, and chemokine ligands (CCL) 2 and 3 [16, 17]. Adoption of the M2 phenotype promotes tissue repair through release of anti-inflammatory cytokines and neurotrophic factors [18].

Sustained inflammatory activation of microglia is implicated as an important mechanism in the progression of many neurodegenerative diseases including multiple sclerosis, stroke, Alzheimer’s disease (AD), and Parkinson’s disease (PD) [19–22]. Experimental models of PD [23] and manganism [4, 5] have often identified the transition of microglia from a resting to activated phenotype prior to overt neuropathology. Cell culture models demonstrate that Mn potentiates inflammatory gene expression in microglia following lipopolysaccharide (LPS)/cytokine treatment through activation of pathways such as nuclear factor kappa B (NF-κB) and mitogen-activated kinase (MAPK) [8, 9]. Removal of microglia or use of antioxidants has shown to reduce neuronal loss, indicating microglial activation may serve as a critical step in mediating neuronal injury during Mn exposure [4, 10].

In addition to neurotoxicity, activated microglia can enhance the activation of adjacent astrocytes by releasing factors such TNF and IL-1β that can further magnify neuronal injury [23, 24]. Microglial responses are often rapid, in contrast to the more delayed activation often seen in astrocytes, suggesting that temporally distinct signaling events are required for a reactive phenotype in each type of glial cell. Underscoring this point, decreased microgliosis *in vivo* is associated with reduced astrocytosis [16]. Despite the known role of microglia-astrocyte crosstalk in AD and PD, these important glial-glial interactions are virtually unknown in manganese neurotoxicity.

Astrocytes serve as the major homeostatic regulator and storage site for Mn in the brain [25] and are a prominent contributor to Mn-stimulated nitric oxide (NO) production through NOS2 [10]. We previously reported that Mn enhances the inductive effects of inflammatory cytokines on astrocyte expression of *Nos2* through stimulation of NF-κB [7] and astrocytes activated by exposure to Mn and inflammatory cytokines induced apoptosis in co-cultured striatal neurons [26]. However, without co-treatment with cytokines, astrocytes are unable to cause neuronal apoptosis in response to Mn treatment [6], indicating that microglia are likely required for initiating neuroinflammatory mechanisms in astrocytes during Mn neurotoxicity. In the current study, we postulated that Mn directly activates microglia and that this could in turn enhance activation of astrocytes through an NF-κB-dependent mechanism. To address this hypothesis, we utilized highly purified cultures of primary microglia and astrocytes to study cell-cell interactions leading to reactive inflammation in glia following exposure to Mn. These studies revealed Mn to be a potent inducer of an inflammatory phenotype in microglia that is essential for activation of astrocytes, suggesting there are critical signaling pathways in glial cells for neuroinflammatory injury from Mn.

## Methods

### Cell culture

Mixed glial cultures from whole brain (excluding the cerebellum and brain stem) were prepared from 1-day-old transgenic mice expressing an enhanced green fluorescent (EGFP) reporter under the control of three cis NF-κB elements (*cis*-NF-κB^EGFP^; C57Bl6/J background [27], generously provided by Dr. Christian Jobin, University of North Carolina at Chapel Hill) using a modification of a previously described method [7, 28, 29]. Briefly, mice were euthanized by decapitation under isofluorane anesthesia, and whole brains were rapidly dissected out and placed into ice-cold minimum essential medium with l-glutamine (MEM; Gibco/Invitrogen, Grand Island, NY). The meninges were removed and tissues completely digested with dispase (1.5 U/mL; Gibco). Dissociated cells were plated onto 100-mm tissue culture plates and kept in minimum essential medium (MEM) supplemented with 10% heat-inactivated FBS (Sigma, St. Louis, MO) and penicillin (0.002 mg/mL), streptomycin (0.002 mg/mL), and neomycin (0.001 mg/mL) antibiotic mixture (PSN). Media was changed every 4–5 days, and cells were maintained at 37 °C and 5% CO_2_ in humidified chambers until cultures were confluent (~14–18 days).

### Purification of astrocytes and microglia

Microglia were purified from astrocytes via column-free magnetic separation using the EasySep Mouse CD11b Positive Selection Kit (Stemcell Technologies, Vancouver, Canada) according to manufacturer instructions and as outlined in [30]. Cells from confluent mixed glial cultures (~14–18 days old) were detached using 0.25% trypsin (Gibco). Trypsin reaction was halted using complete MEM, and remaining cells were removed using a cell lifter. Harvested cells were gently triturated and passed through a 70-μm cell strainer to remove any cell aggregates. The cell mixture was then centrifuged and resuspended at 1 × 10^8^ cells/mL in calcium- and magnesium-free phosphate-buffered saline containing 2% FBS and 1 mM EDTA. Cells were transferred to a 5-mL round-bottom tube and were incubated at room temperature with the CD11b-Phycoerythrin (PE) monoclonal antibody (50 μL/mL) for 15 min then EasySep PE-Selection Cocktail (70 μL/mL) for 15 min followed by a 10-min incubation with dextran-coated EasySep magnetic nanoparticles (50 μL/mL). Cell suspension was brought up to 2.5 mL of media, gently mixed, and placed in the EasySep magnet for 5 min to isolate immune-linked cells. After 5 min, the tube was inverted and the cell solution of un-labeled cells was collected while labeled cells remained in the tube. The solution remaining in the tube was resuspended in another 2.5 mL of recommended media and placed in the magnet. This process was repeated for a total of five extractions. The purified microglia in the positive fraction and purified astrocytes in the final three negative fractions were resuspended in complete MEM and seeded onto tissue culture plates. Purified astrocytes and microglia were utilized in experiments within a week of purification.

### Flow cytometry

The estimated percent of glia in mixed glial, microglial, and astrocyte cultures were determined by immunophenotying using direct labeling with anti-GLAST-PE (Miltenyi Biotec, San Diego, CA), anti-Cd11b-FITC (BD Biosciences), anti-CD11b-PE (Stemcell Technologies), and anti-GLAST-488 (Novus Biologicals, Littleton, CO) followed by flow cytometric analysis. Cells were counted using a Bio-Rad TC10 automated cell counter, and 1 × 10^6^ cells/mL were resuspended in 100 μL of incubation buffer (PBS with 0.05% bovine serum albumin). Mixed glial cultures were labeled using the mouse anti-GLAST-PE (20 μg/mL) and mouse anti-CD11b-FITC (10 μg/mL) at room temperature for 1 h. Microglia cultures were incubated with CD11b-PE according to manufacturer instructions while astrocyte cultures were incubated with rabbit polyclonal anti-GLAST-488 (10 μg/mL) at room temperature for 1 h. After labeling, the cells were washed twice in incubation buffer and resuspended at a final volume of 500 μL of PBS and stored at 37 °C until analysis. Flow cytometry was performed on a Beckman Coulter CyAn ADP flow cytometer operated with Summit software for data collection at Colorado State University’s Flow Cytometry Core Facility. All further data analysis was done utilizing FlowJo software (version 10.1; FlowJo, Ashland, OR).

### Immunofluorescence

Mixed glial cultures and purified microglia and astrocytes obtained from magnetic separation were seeded onto serum-coated 12-mm glass coverslips at a density of 1 × 10^5^ cells/well and allowed to adhere for 48 h. For all experiments except for determination of purity, microglia were treated for 24 h with either saline or 100 μM MnCl_2_ for 24 h. Cells were fixed using methanol, washed in PBS, and then blocked in 1% bovine serum albumin (w/v) in PBS for 1 h. Cells were incubated overnight at 4 °C in primary antibodies for ionized binding adaptor protein-1 (IBA-1; 1:50; Wako, Osaka, Japan) and glial fibrillary acidic protein (GFAP; 1:500; Sigma) for purity experiments, IBA-1 for morphology experiments, and NOS2 (1:100; BD Biosciences, San Jose, CA) for expression analysis. After rinsing in PBS, cells were incubated for 1 h at room temperature in Alexa Fluor 488- and/or Alexa Fluor 647-conjugated secondary antibodies (1:500; Invitrogen, Carlsbad, CA) and then mounted in medium containing 4′,6-diamidino-2-phenylindole dihydrochloride (DAPI) to detect cell nuclei. Images were acquired using a ×20 or ×40 air plan apochromatic objectives on a Zeiss Axiovert 200M inverted fluorescence microscope (Carl Zeiss, Inc., Thornwood, NY) equipped with a Hamamatsu ORCA-ER-cooled charge-coupled device camera (Hamamatsu Photonics, Hamamatsu City, Japan).

Determination of purity was performed by counting the number of IBA-1- and GFAP-positive cells in at least six fields in at least three separate coverslips. This was performed for every purification experiment.

NOS2 fluorescence intensity was determined using SlideBook software (Version 5.0, Intelligent Imaging Innovations, Denver, CO). Acquired images were masked for FITC, and the sum intensity was reported per field then normalized for the number of somas. This was performed in at least six fields in at least three separate coverslips per treatment.

### Gene expression

Purified microglia were seeded onto six-well tissue culture plates at a density of 3 × 10^5^ cells/well and treated with saline (equal volume as treated, 1 μL/mL), to account for changes in osmolarity when treating cells with MnCl_2_, or MnCl_2_ at 10, 30, or 100 μM from 2 to 24 h. Doses of MnCl_2_ were chosen to range from normal to three to five times physiological levels based on previous research indicating that oral exposure in humans and rodents can result in upwards of two to six fold normal physiological concentrations (normal ranges between 10 and 30 μM) [31–34]. Astrocytes were seeded and treated as described below. RNA was isolated from glia utilizing the RNeasy Mini Kit (QIAGEN, Valencia, CA) with purity and concentration confirmed using a NanoDrop ND-1000 spectrophotometer (NanoDrop Technologies, Wilmington, DE). Two hundred fifty nanograms of RNA (microglia) and 500 ng of RNA (astrocytes) were used as a template for reverse transcriptase reactions using the iScript RT kit (Bio-Rad, Hercules, CA). cDNA was mixed with SYBR Green (Bio-Rad, Hercules, CA). Primer pairs are listed in Table 1. The 2^−ΔΔ^CT method [35] was used to determine fold expression with normalization to β-actin or hypoxanthine-guanine phosphoribosyl transferase 1 (HPRT) expression.

**Table 1:** qPCR primer sequences

Gene	Accession number	Forward primer (5′-3′)	Reverse primer (5′-3′)	Reference
*β-actin*	NM_00793.3	GCTCTCCTATGTTGCTCTAG	CGCTCCTTGCCAATACTC	^a^
*Bdnf*	NM_001048139.1	TTACCTGGATGCCGCAAACAT	TGACCCACTCGCTAATACTGTC	^b^
*Caspase 1*	NM_009807.2	ATGAATACAACCACTCGTACAC	ATCCTCCAGCAGCAACTTC	^c^
*Ccl2*	NM_011331.2	TTAAAAACCTGGATCGGAACCAA	GCATTAGCTTCAGATTTACGGGT	^d^
*Ccl5*	NM-013653.3	GCTGCTTTGCCTACCTCTCC	TCGAGTGACAAACACGACTGC	^d^
*Cd16*	NM_010188.4	TTTGGACACCCAGATGTTTCAG	GTCTTCCTTGAGCACCTGGATC	^e^
*Cd206*	NM_008625.1	TCTTTGCCTTTCCCAGTCTCC	TGACACCCAGCGGAATTTC	^e^
*Cd32*	NM_010187.2	AATCCTGCCGTTCCTACTGATC	GTGTCACCGTGTCTTCCTTGAG	^e^
*Cd86*	NM-019388	TTGTGTGTGTTCTGGAAACGGAG	AACTTAGAGGCTGTGTTGCTGGG	^e^
*Igf-1*	NM_010512.4	AAAGCAGCCCGCTCTATCC	CTTCTGAGTCTTGGGCATGTCA	^b^
*Il-1β*	NM_008361.3	GCAGCAGCACATCAACAAAG	CACGGGAAAGACACAGGTAG	^c^
*Il-6*	NM_031168.1	GACAACTTTGGCATTGTGG	ATGCAGGGATGATGTTCTG	^b^
*Nos2*	NM_010927.2	TCACGCTTGGG CTTGTT	CAGGTCACTTTGGTAGGATTTG	^a^
*Tnf*	NM_013693.3	GTTGCCTGATTCTTGCTTCTG	GCCACCACTTGCTCCTAC	^c^

^a^Sequences taken from [39]

^b^Sequences obtained from PrimerBank

^c^Primers designed using NCBI/Primer-BLAST

^d^Sequences taken from [39]

^e^Sequences taken from [18]

### Microglial morphology

Microglia were prepared and imaged as described above for immunofluorescence. Acquired ×20 images were analyzed using Fiji software for changes in microglia morphology via a method reported in [36]. Briefly, images were enhanced to visualize all microglia processes via standard background subtraction (50 pixels with sliding parabola option) and despeckled to eliminate single-pixel background fluorescence. The resulting images were converted to a binary image and then skeletonized. The AnalyzeSkeleton plugin (http://imagejdocu.tudor.lu/) was utilized to analyze all skeletonized images and to report the number of end point voxels, number of branches, maximum branch length, and average branch length. These data points were utilized to assess microglia morphology and normalized to the number of somas per field. At least six fields were analyzed in at least three separate coverslips for each treatment.

### Microglia-conditioned media experiments

Microglia were seeded onto six-well tissue culture plates at 3 × 10^5^ cells/well and treated with saline or 100 μM MnCl_2_ for 24 h. Microglia-conditioned media (MCM) was pooled per treatment and centrifuged at 800×*g* for 10 min to remove detached cells. Media was placed onto astrocytes seeded in six-well tissue culture plates at 6 × 10^5^ cells/well with no additional MnCl_2_ added for 24 h. The ratio of 1:2 microglia to astrocytes was utilized as astrocytes are known to comprise 20–40% of cells in a given brain region while microglia compose 5–15% with higher levels of microglia reported in the basal ganglia [37, 38]. As a comparison, astrocytes plated at the same density were treated with saline or 100 μM MnCl_2_ concurrent to astrocytes treated with MCM. RNA was extracted as cited above and analyzed for changes in gene expression.

To inhibit NF-κB signaling in microglia, cells seeded in six-well plates were treated with 5 μM Bay 11-7082 (Bay11; Sigma) or the vehicle dimethyl sulfoxide (DMSO; Sigma) at 0.05% in complete media for 3 h. Exposure to Bay11 or DMSO for 3 h had no effect on cell viability while sufficiently blocking NF-κB-regulated gene expression. Bay11 was chosen due to its established ability to inhibit of NF-κB including in primary microglia [39, 40]. Media was completely exchanged, and microglia were then treated per standard MCM protocol with either saline or 100 μM MnCl_2_.

For knockdown of TNF, RNAi oligos obtained through Integrated DNA Technologies (IDT, Coralville, IA) were transfected into microglia using the *Trans*IT-X2 delivery system (Mirus Bio, Madison, WI) 48 h prior to MnCl_2_ treatment. RNAi duplexes were designed against common *Tnf* sequence (*Tnf* siRNA) and validated using a dose-response assay of the suspended oligos (12.5 to 25 μM) in lipopolysaccharide (1 μg/mL)-treated BV2 microglial cell line using a standard scrambled dicer-substrate RNA (Scr siRNA) as a control. The *Tnf* siRNA sequences were GGAUGAGAAGUUCCCAAAUGGCCTC; UCCCUACUCUUCAAGGGUUUACCGGAG, and the Scr siRNA sequences were CUAGGUUGAAGAUGUUAUAGGCACT; AGUGCCUAUAACAUCUUCAACCUAGAA.

### Microglia-astrocyte co-culture

Purified microglia were seeded onto permeable cell culture inserts (BD Biosciences) at 3 × 10^5^ cells/well and astrocytes onto six-well tissue culture plates at 5 × 10^5^ cells/well and allowed to adhere for 48 h. Cell media was changed 24 h prior to treatment. Microglia inserts were placed into six-well culture plates seeded with astrocytes, and both were treated with either saline or 100 μM MnCl_2_ concurrently for 24 h. RNA was then isolated from astrocytes as detailed above and analyzed for changes in gene expression.

### Measurement of Mn uptake in microglia

Purified microglia were seeded onto 96-well tissue culture plates at 2 × 10^4^ cells/well and exposed to saline or MnCl_2_ at 10, 30, or 100 μM for 24 h in complete MEM. Cellular uptake of Mn was then performed using a previously described cellular fura-2 manganese extraction assay (CFMEA) that calculates cellular Mn based on fura-2 quenching [41]. Briefly, treated cells were washed with phosphate-buffered saline (PBS) and extracted in PBS + 0.1% Triton X (PTX) with 2 μM fura-2 (Sigma-Aldrich) for 1 h at 37 °C. Fura-2 fluorescence was measured at the Ca^2+^ isosbestic point of Ex_360_ (bandwidth filter ±25 nm) with a BioTek Cytation 3 Cell Imaging Multi-Mode Reader using Gen5 imaging software (version 2.5) and top read settings. Average raw fluorescence signal values were converted to percent maximal fluorescence (%_MAX_) after background subtraction. Cellular Mn concentrations were then calculated from %_MAX_ using non-linear regression analysis in Microsoft Excel from saturation binding standard curves generated from a cell-free system consisting of stock Mn standards (0–100 mM) in fura-2 in PTX imaged concurrently with treated cells. Power curves (Mn concentration = A(%_MAX_)^B^) were used for %_MAX_ values less than 50% and logarithmic curves (Mn concentration = A ln(%_MAX_) + B) for values greater than 50%.

### Measurement of Mn in cell culture media

Media samples (500 μL) were collected from saline- and 100 μM MnCl_2_-treated microglia at the start of and 24 h after treatment. Media was stored at −80 °C until analyzed. Metals were complexed in the samples using hydrochloric acid then brought up to a final volume of 1 mL using Milli-Q water. Analysis was performed by inductively coupled plasma mass spectrometry (ICP-MS) on a PerkinElmer ELAN DRC II instrument (PerkinElmer, Waltham, MA) at the Center for Environmental Medicine Analytical Core at Colorado State University. Media blanks underwent the same processing to ensure low levels of Mn in complete MEM. At least three samples were analyzed per treatment group.

### Measurement of cytokines in microglia-conditioned media

Media was sampled from microglia during MCM experiments prior to application on astrocytes and stored at −80 °C. Stored media was thawed, and cytokines were measured using a mouse 14-plex ELISA (Q-Plex^TM^ Mouse Cytokine Arrays, Quansys Biosciences, Logan, UT) according to manufacturer instructions and imaged on a ChemiDoc XRS (Life Science Research, Hercules, CA) to capture images. Levels of cytokines and chemokines were calculated from standard curves using Q-View imaging software (Quansys Biosciences). To measure TNF levels in media, a Ready-SET-Go!® TNF single-plex ELISA (eBioscience Inc., San Diego, CA) was utilized according to manufacturer instructions.

### Statistical analyses

All statistical analysis was performed using Prism software (version 6.0; GraphPad Software, Inc., San Diego, CA) with a Student’s *t* test utilized for comparison of two means and a one-way analysis of variance (ANOVA) followed by a Tukey-Kramer multiple comparison post hoc test for comparison of three or more means. Two-way ANOVA followed by a Tukey-Kramer multiple comparison post hoc test was used for comparison of three or more means and two different variables in analyzing astrocyte responses in co-culture and conditioned media experiments. Independent variables for two-way ANOVA were defined as treatment (saline versus Mn) and microglial presence (Mn alone vs. presence of microglia/MCM). Statistical significance was defined as a *p* value <0.05.

## Results

### Purity of glia cultures

**Fig. 1:**
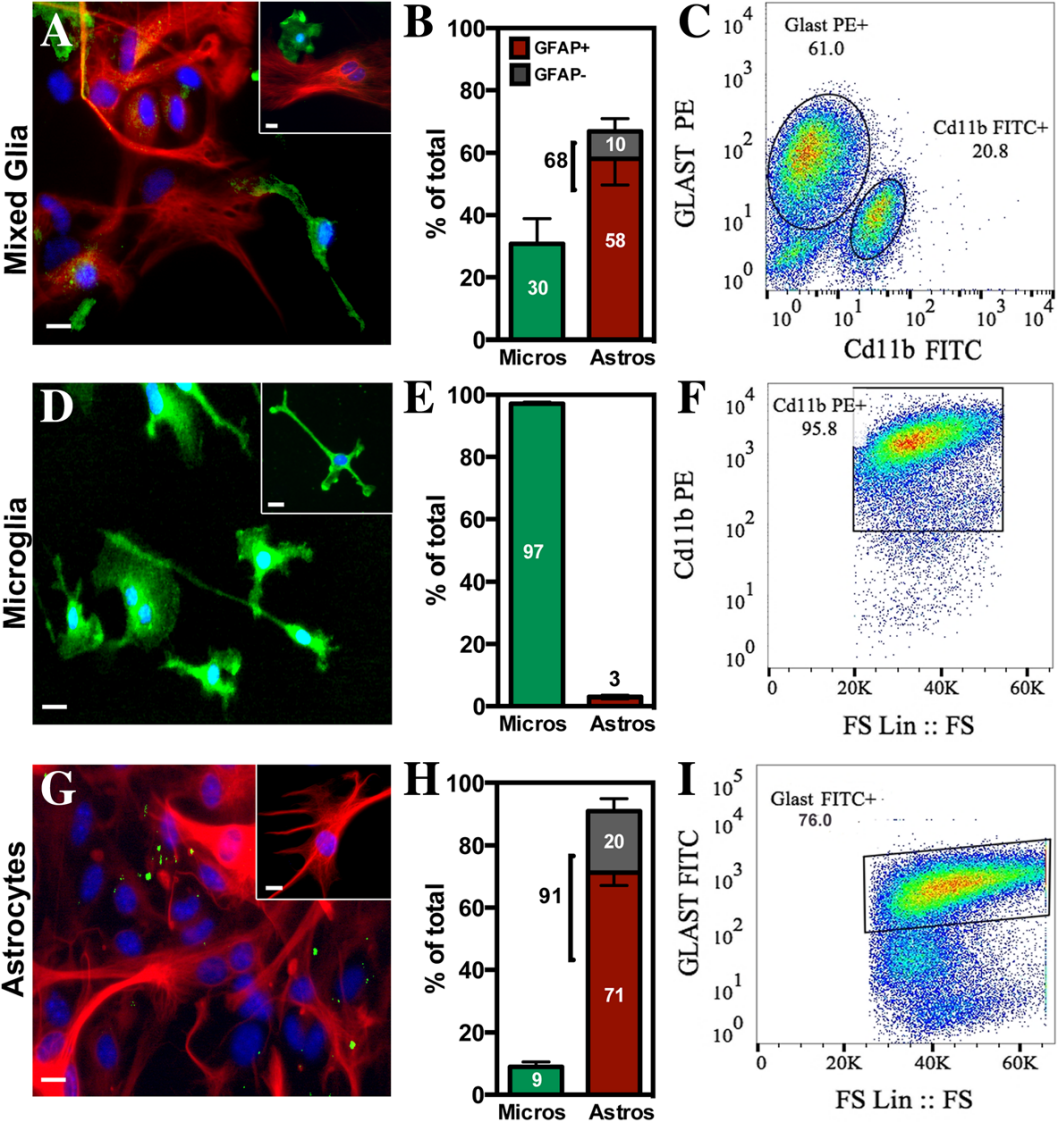
Column-free immunomagnetic separation generates highly pure cultures of microglia. Mixed glia (**a**–**c**), microglial (**d**–**f**), and astrocyte (**g**–**i**) cultures were assessed for total glia composition via immunofluorescence for GFAP-positive (*red*) and IBA-1-positive (*green*) cells or via flow cytometry for Cd11b and GLAST. Representative ×20 images of mixed glia (**a**), microglial (**b**), and astrocyte (**c**) cultures with a ×40 *insert* showing GFAP (*red*), IBA-1 (*green*), and DAPI (*blue*). *Scale bars* = 10 μm. Quantitative counts were determined for the number of glia present in mixed glia (**b**), microglia (**e**), and astrocyte (**h**) cultures both by positive immunoreactivity (*colored*) or by consistent morphology in the absence of positive staining (*gray*). Data are presented as mean percent of total cells per field ± SEM. Flow cytometry scatter plots showing the percentage of Cd11b- or GLAST-positive cells for mixed glia (**c**), microglia (**f**), or astrocyte (**i**) cultures

The purity of microglia and astrocytes isolated from mixed glial cultures via a column-free immune-magnetic method was assessed by co-immunofluorescence and flow cytometry (Fig. 1). Representative images of the glial composition of each culture are shown in Fig. 1a, d, g. Quantitative counts for the percent of total cells immunolabeled for IBA-1 or GFAP revealed that mixed cultures contained roughly 30% IBA-1+ cells (microglia) and 68% astrocytes (Fig. 1b). Not all astrocytes stained positive for GFAP (10% did not), and remaining counts were determined via morphology by bright-field microscopy. Following purification, microglial cultures contained 97% IBA-1+ cells (Fig. 1e) and astrocyte cultures contained 91% astrocytes (71% GFAP+, 20% GFAP−; Fig. 1h). To further determine the distribution of glial cell types in immunopurified fractions, cultures of mixed glia, microglia, and astrocytes were examined by flow cytometry using distinct immunolabeling markers for microglia (Cd11b) and astrocytes (GLAST/SLC1A3). The percentage of each glial cell type determined by flow cytometry in mixed glial cultures was ~21% Cd11B+ cells (microglia) and 61% GLAST+ cells (Fig. 1c), microglia cultures was 96% Cd11b+ cells (Fig. 1f), and astrocyte cultures was 76% GLAST+ cells (Fig. 1i), similar to the distribution determined by immunofluorescence microscopy.

### Mn induces an activated, inflammatory phenotype in microglia

**Fig. 2:**
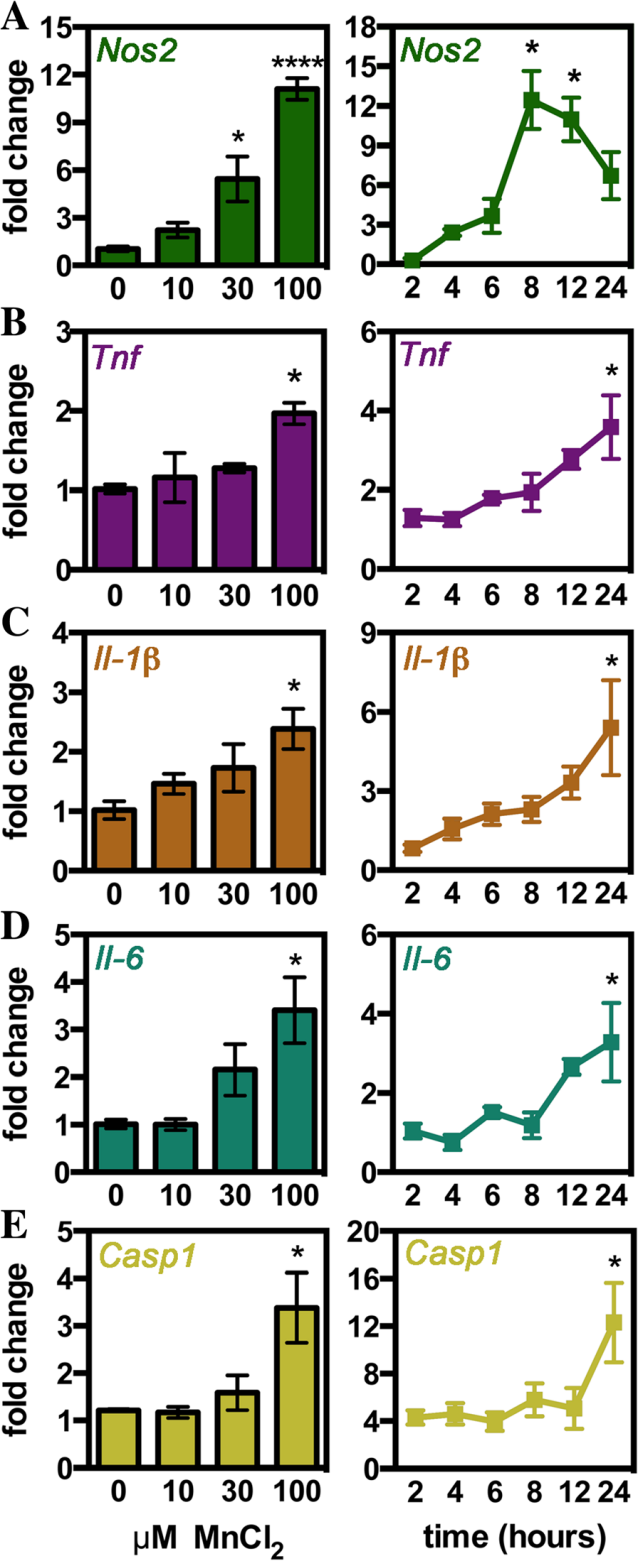
Manganese directly induces dose- and time-dependent expression of inflammatory genes in microglia. Purified primary microglia were treated with increasing doses of MnCl_2_ (0–100 μM) and over time (at 100 μM MnCl_2_) to determine dose-dependent (*left panels*) and time-dependent (*right panels*) effects of Mn on *Nos2* (**a**), *Tnf* (**b**), *Il-1β* (**c**), *Il-6* (**d**), and *caspase 1* (**e**) expression. Data are presented as fold change in mRNA ± SEM (one-way ANOVA; *asterisks* indicate significance from control with *p < 0.05 and ***p < 0.001)

To identify the extent of microglial activation in response to Mn in the absence of astrocytes, the expression of multiple inflammatory genes was determined in immunopurified cultures of primary microglia exposed to various concentrations of Mn (Fig. 2). Treatment of primary microglia for 24 h to 0, 10, 30, or 100 μM MnCl_2_ resulted in dose-dependent upregulation of *Nos2*, *Il-6*, *Il-1β*, *Tnf*, and *caspase 1*; however, only *Nos2* was upregulated significantly at a dose lower than 100 μM MnCl_2_. Assessment of Mn treatment over time revealed that Mn time-dependently activates microglia with *Nos2* upregulated early at 6 h of treatment while cytokine and *caspase 1* expression is not significant from control until 24 h. Based on these results, future treatments of microglia were conducted with 100 μM MnCl_2_ for 24 h.

**Fig. 3:**
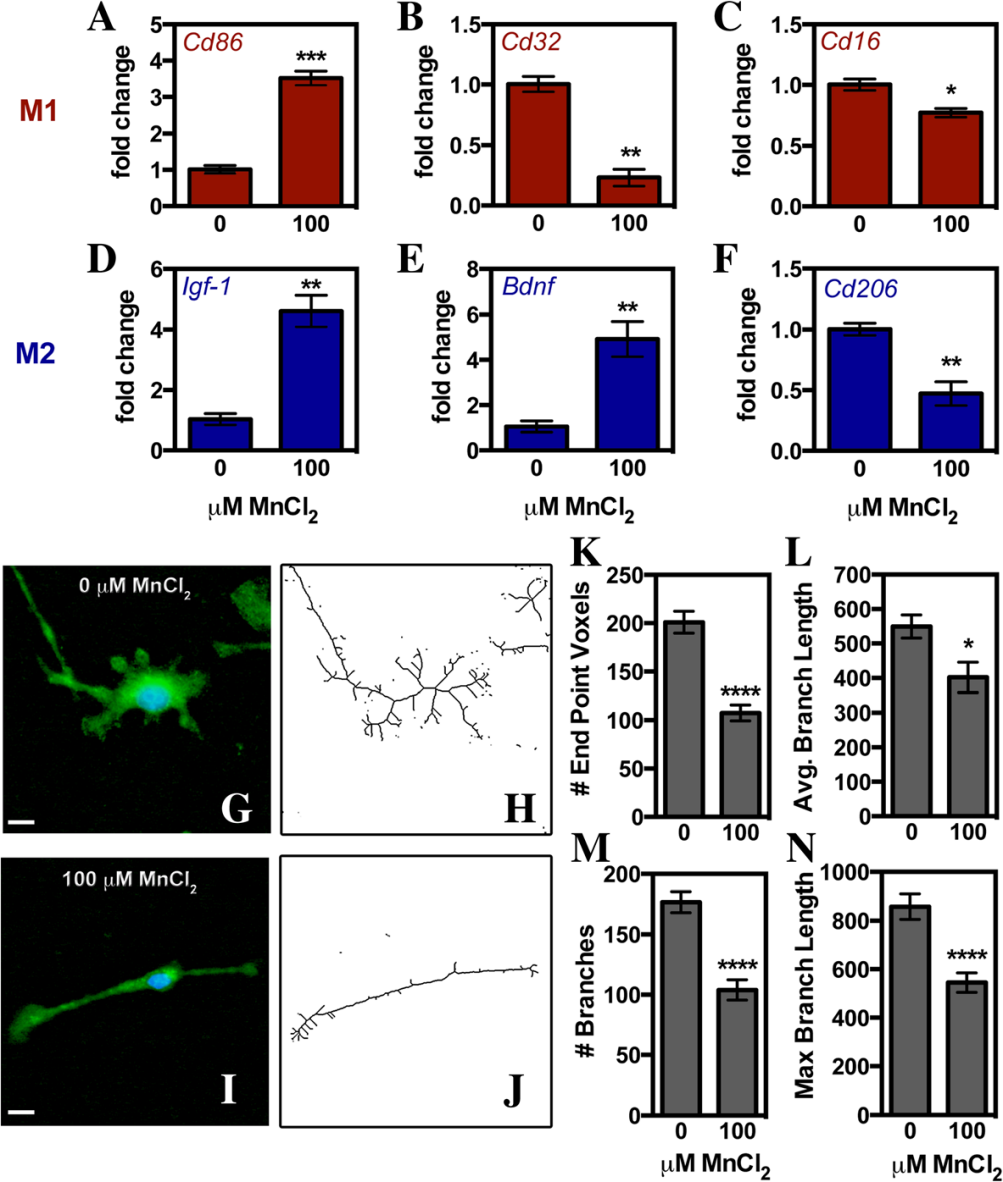
Manganese causes a mixed inflammatory phenotype in microglia. Microglial phenotype after 24-h treatment with 100 μM MnCl_2_ was assessed via qPCR measurement of M1 (**a**–**c**, *red*) and M2 (**d**–**f**, *blue*) genes or by analyzing changes in morphology via immunofluorescence (**g**–**n**). M1 genes analyzed included *Cd86* (**a**), *Cd32* (**b**), and *Cd16* (**c**). M2 genes analyzed included *Igf-1* (**d**), *Bdnf* (**e**), and *Cd206* (**f**). Data are presented as the mean mRNA fold change ± SEM (Student’s *t* test; *p < 0.05, **p < 0.01, and ***p < 0.0001). Representative ×40 images used to assess morphology from control (**g**) and 100 μM MnCl_2_-treated (**i**) microglia with IBA-1 (*green*) and DAPI (*blue*). These images were converted to binary then skeletonized (**h**–**j**) to assess changes in morphology including the number of end point voxels (**k**), average branch length (**l**), number of branches (**m**), and maximum branch length (**n**). Data is presented as indicated value ± SEM (Student’s *t* test; *p < 0.05 and ****p < 0.0001)

Microglia are a myeloid-derived cell, and recent evidence suggests that they can express two different phenotypes: an inflammatory M1 phenotype and an anti-inflammatory M2 phenotype [15, 18]. Assessment of Mn-treated microglia for expression of prototypical M1 markers *cluster of differentiation* (*Cd*) *86*, *Cd32*, and *Cd16* revealed mixed results with significant upregulation of *Cd86* and downregulation of *Cd32* and *Cd16* (Fig. 3a–c). Similar mixed results were seen when evaluating classical M2 genes with brain-derived neurotrophic factor (*Bdnf*) and insulin-like growth factor-1 (*Igf-1*) upregulated while *Cd206* was significantly reduced in Mn-treated microglia (Fig. 3d)–f).

Alterations in cellular morphology are another way to measure microglial activation following stress or inflammatory insult. Resting microglia have a ramified phenotype and then transition to a more rod-like and finally amoeboid shape upon activation [42]. Morphological changes in purified microglia exposed to either 0 or 100 μM MnCl_2_ for 24 h were quantified via immunofluorescence imaging of IBA-1 (Fig. 3g, i) and conversion of fluorescent images to a skeletonized image (Fig. 3h, j). Analysis of skeletonized images using Fiji showed significant de-ramification of microglia exposed to Mn, indicated by a reduction in the number of end point voxels (Fig. 3k), average branch length (Fig. 3l), number of branches (Fig. 3m), and the maximum branch length (Fig. 3n). The majority of microglia adopted a rod-like phenotype following Mn exposure with only a small percentage of microglia transitioning fully to an amoeboid shape.

### Mn-activated microglia mediate neuroinflammatory activation of astrocytes

**Fig. 4:**
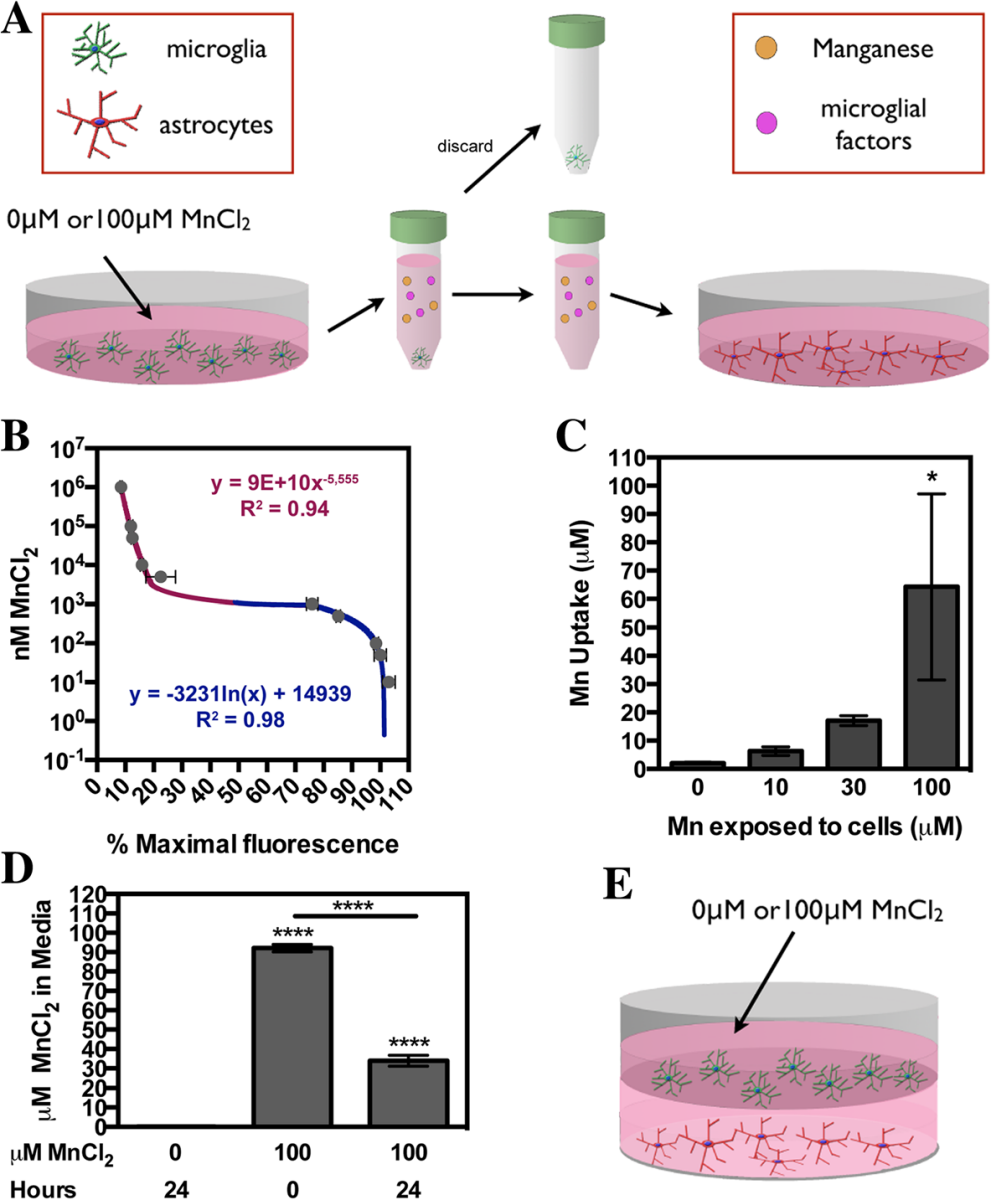
Microglia uptake 70% of Mn present in media. **a** Schematic diagram outlining the procedure for microglia-conditioned media (MCM) experiments. The amount of Mn remaining in MCM media was assessed via microglia cell uptake via cellular fura-2 manganese extraction assay (CFMEA) (**b**, **c**) or by measuring Mn levels in media (**d**). **b** Cell-free Mn-fura-2 saturation binding standard curve generated to calculate Mn uptake in microglia over a 24-h period. Data is represented as mean levels ± SD at %_MAX_ (*x-axis*) and log10 scale of MnCl_2_ concentration (*y-axis*) with power curve (*red line*) used for %_MAX_ less than 50% and logarithmic curve (*blue line*) used for %_MAX_ greater than 50%. **c** The calculated amount of Mn uptake (*y-axis*) per MCM experiment measured via CFMEA in cultured microglia cells exposed to increasing doses of MnCl_2_ (0–100 μM; *x-axis*) over 24 h. Data is represented as mean Mn uptake per 2.5 × 10^5^ cells ± SEM (one-way ANOVA; *asterisk* indicates significance from control; *p < 0.05). **d** The levels of Mn in MCM were measured via ICP-MS at time 0 and 24 h post treatment. Data are presented as the mean micromolar concentration of Mn ± SEM (one-way ANOVA; *asterisks* indicate significance from control; ****p < 0.0001). **e** Schematic diagraming procedure for microglia-astrocyte co-culture experiments

To assess the ability of Mn-activated microglia to modulate astrocyte activation, we treated astrocytes either with microglia-conditioned media (MCM) from Mn-treated microglia (Fig. 4a) or directly with Mn in the presence of microglia using a co-culture model (Fig. 4e). Because the kinetics of Mn transport into microglia is not well characterized, we first determined the concentration of Mn remaining in the media after 24 h of incubation with microglia to determine the amount of Mn remaining in MCM that was applied to astrocytes as no additional MnCl_2_ was added. This was determined by measuring microglial uptake via quenching of fura-2 from intracellular extractions using the described CFMEA technique (Fig. 4c) and by measuring Mn in MCM via ICP-MS (Fig. 4d). Quantification of Mn uptake was calculated based on non-linear regression equations obtained from fura-2 quenching in a cell-free system using standard concentrations of Mn (Fig. 4b). Over the course of 24 h, microglia exposed to 0, 10, 30, or 100 μM MnCl_2_ took up roughly 60–70% of the exposed Mn (Fig. 4c). This uptake of about 70 μM of Mn correlated to the measured amount of Mn remaining in MCM prior to application on astrocytes (~30 μM; Fig. 4d).

**Fig. 5:**
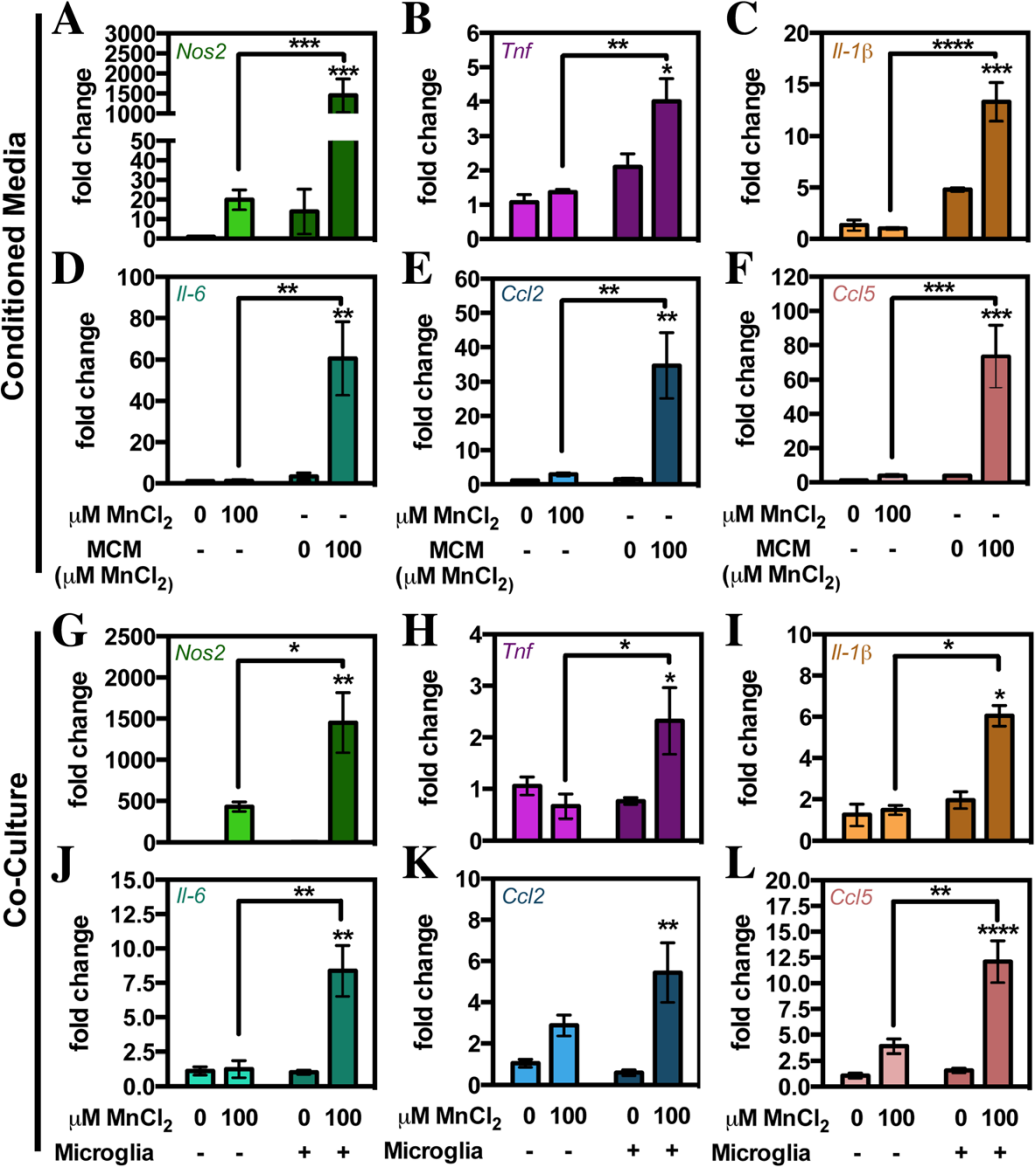
Presence of microglia or microglial-derived factors amplify Mn-dependent activation of astrocytes. Levels of inflammatory gene expression were determined by qPCR in astrocytes following direct treatment with 0 or 100 μM MnCl_2_ versus treatment with Mn from MCM (**a**–**c**) or when co-cultured with microglia (**d**–**f**). **a** Upregulation of astrocyte inflammatory gene expression including *Nos2* (**a**, **d**), *Tnf* (**b**, **e**), *Il-1β* (**c**, **f**), *Il-6* (**d**, **j**), *Ccl2* (**e**, **k**), and *Ccl5* (**f**, **l**) required the presence of either microglia-derived factors (MCM) or the presence of microglia (co-culture) for significant induction. Data are presented as the mean mRNA fold change ± SEM (two-way ANOVA; *asterisks above bars* indicate significance from control within a group designated by *different color shading*; *p < 0.05, **p < 0.01, ***p < 0.001, and ****p < 0.0001)

Upon activation, astrocytes rapidly increase the expression of a variety of classical inflammatory genes including chemokines, cytokines, and *Nos2* [22]. To measure the effect of microglia or microglia-derived factors on astrocyte expression of inflammatory genes, qPCR was performed on astrocytes that were exposed to MCM or co-cultured with microglia and compared to the effect of direct treatment with Mn alone (Fig. 5). Gene expression analysis revealed marked differences in astrocytes treated with Mn in the absence of microglia or MCM compared to those treated with Mn in the presence of either MCM or co-cultured microglia. Treatment of astrocytes with 100 μM MnCl_2_ resulted in a large, albeit not significant, increase in the fold expression of *Nos2* but did not alter levels of astrocyte expression of *Tnf*, *Il-1β*, *Il-6*, *Ccl2*, or *Ccl5*. Exposure to MCM from saline-exposed microglia caused a slight, but not significant, increase in astrocyte inflammatory gene expression of *Nos2*, *Tnf*, and *Il-1β* as compared to saline-only-treated astrocytes. However, treatment with MCM from Mn-exposed microglia potentiated expression of *Nos2* (Fig. 5a) and significantly increased astrocyte expression of *Tnf* (Fig. 5b), *Il-1β* (Fig. 5c), *Il-6* (Fig. 5d), *Ccl2* (Fig. 5e), and *Ccl5* (Fig. 5f). Astrocytes co-cultured with microglia showed similar amplification of inflammatory gene expression following treatment with Mn, with increased levels of mRNA detected for *Nos2* (Fig. 5g), *Tnf* (Fig. 5h), *Il-1β* (Fig. 5i), *Il-6* (Fig. 5j), *Ccl2* (Fig. 5k), or *Ccl5* (Fig. 5l), as determined by two-way ANOVA. The magnitude of inflammatory gene expression was substantially different in the two glial treatment paradigms, with much larger changes in astrocyte gene expression in the presence of MCM versus co-culturing for all genes assessed. Overall, inflammatory gene expression in astrocytes was dramatically enhanced both by the presence of co-cultured microglia and by MCM compared to direct treatment with Mn in the absence of microglia or MCM.

### Mn-treated microglia release a variety of inflammatory factors into media

**Fig. 6:**
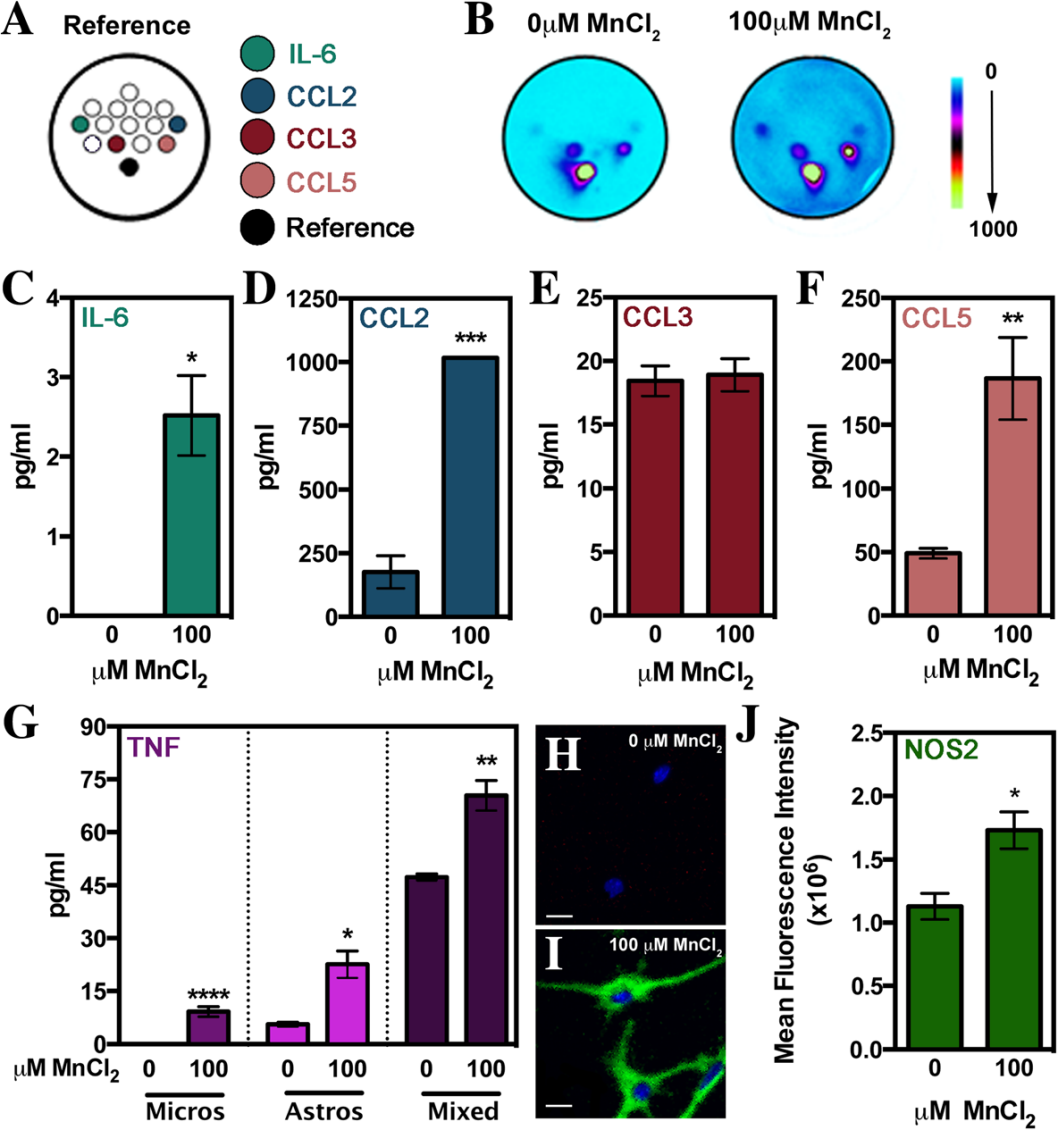
Mn-exposed microglia release a variety of proinflammatory mediators. Multi-plex or single-plex ELISA was utilized to assess the levels of cytokines/chemokines in sampled media from 0 or 100 μM MnCl_2_-treated microglia. **a** Representative image from the Quansys mouse 14-plex array indicating location of noted chemokines and cytokines. **b** Representative heat maps of Quansys multi-plex ELISA results of media from 0 or 100 μM MnCl_2_-treated microglia with dot locations correlating to **a**. Intensity is represented by no expression (*blue*) to increasing expression (*yellow*). Levels of IL-6 (**c**), CCL2 (**d**), CCL3 (**e**), CCL5 (**f**), and TNF (**g**) were calculated based on standard curves generated during experiments. Data is represented by mean concentration (pg/mL) ± SEM (Student’s *t* test; *p < 0.05, **p < 0.01, and ***p < 0.001). NOS2 levels (*green*) were assessed via immunofluorescence in 0 (**h**) or 100 μM (**i**) MnCl_2_-treated microglia and quantified by measuring the mean fluorescence intensity per cell (**j**). Data is represented by mean fluorescence per cell per field ± SEM (Student’s *t* test; *p < 0.05). *Blue* = DAPI; *scale bar* = 10 μm

Once it was determined that microglia potentiated inflammatory activation of Mn-exposed astrocytes, we sought to identify putative signaling factors that could be involved in microglia-astrocyte communication by measuring signaling proteins released by microglia via multi-plex and single-plex ELISA (Fig. 6). Using a multi-plex ELISA, we simultaneously assessed the protein levels of IL-6, CCL2, CCL3, and CCL5 in media from microglia exposed to saline or 100 μM MnCl_2_ for 24 h (Fig. 5a). As represented by the heat map images in Fig. 5b, protein quantification revealed significant increases in microglial release of the cytokine IL-6 (Fig. 6c) and chemokines CCL2 (Fig. 6d) and CCL5 (Fig. 6f) but not in CCL3 (Fig. 6e) or TNF (not shown). However, due to the lower sensitivity of the multi-plex ELISA for TNF, we also analyzed TNF in cellular supernatants using a separate, highly sensitive single-plex ELISA (Fig. 6g). For this assay, TNF was measured from media acquired from microglia, astrocytes, or co-culture (mixed glia) treated with either saline or 100 μM MnCl_2_ for 24 h (Fig. 6g). TNF release into media was significantly increased in microglia or astrocytes treated with Mn alone; however, the most significant release of TNF occurred in mixed glial populations containing both astrocytes and microglia.

During neuroinflammation, the largest source of NO is produced by activated glia expressing the inducible form of nitric oxide synthase (iNOS/NOS2). Once produced, NO diffuses freely across cellular membranes acting as both a source of oxidative stress through peroxynitrite formation and a signaling molecule known to modulate a variety of genes through activation of soluble guanlyl cyclase [43]. NOS2 protein expression was measured in saline-treated (Fig. 6h) or 100 μM MnCl_2_-treated (Fig. 6i) microglia via immunofluorescence. Measurement of NOS2 fluorescence intensity (Fig. 6j) revealed significant increases in NOS2 protein in Mn-treated microglia compared to controls.

### NF-κB signaling in Mn-treated microglia is required for glial crosstalk with astrocytes

**Fig. 7:**
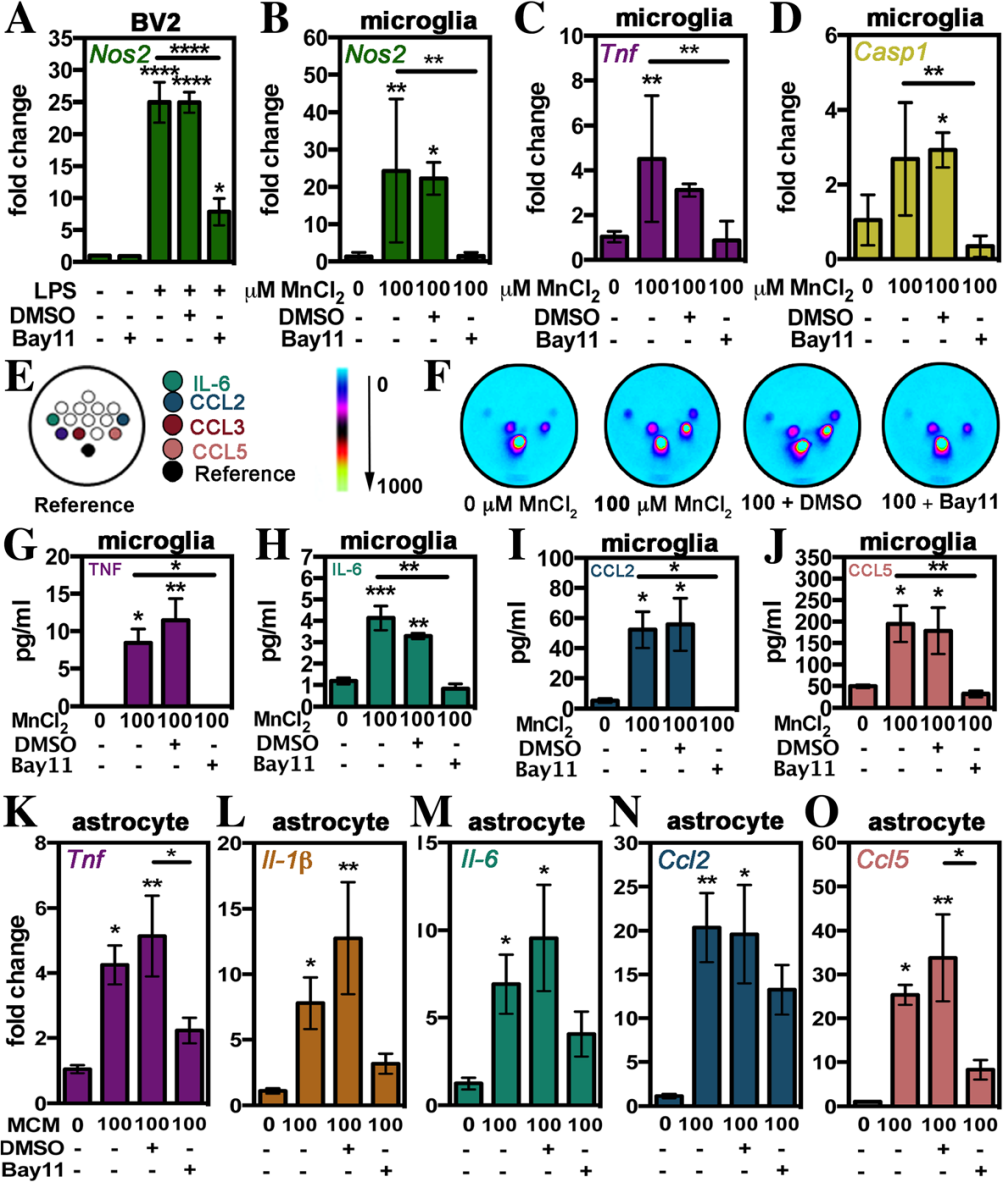
Glial crosstalk in Mn toxicity relies on NF-κB signaling in microglia. Prior to use in MCM experiments, microglia or BV2 microglia cells were pretreated with the NF-κB inhibitor Bay11 or DMSO. **a** Suppression of NF-κB-driven expression of *Nos2* was assessed in BV2 microglial cells treated with lipopolysaccharide (1 μg/mL) for 24 h. Suppression of NF-κB-regulated inflammatory genes in microglia was measured via qPCR for *Nos2* (**b**), *Tnf* (**c**), and *caspase 1* (**d**). Representative image (**e**) from the Quansys mouse 14-plex array indicating location of noted chemokines and cytokines. (**f**) Representative heat maps of Quansys multi-plex ELISA results of media from 0 or 100 μM MnCl_2_-treated microglia in combination with DMSO or Bay11 with dot locations correlating to **e**. Intensity is represented by no expression (*blue*) to increasing expression (*yellow*). Levels of TNF (**g**), IL-6 (**h**), CCL2 (**i**), and CCL5 (**j**) were calculated based on standard curves generated during experiments. Levels of inflammatory gene expression in astrocytes treated with MCM versus MCM of microglia treated with DMSO or Bay11 were determined via qPCR for *Tnf* (K), *Il-1β* (**l**), *Il-6* (**m**), *Ccl2* (**n**), and *Ccl5* (**o**). Expression data is represented by mRNA fold change ± SEM while ELISA data is represented by mean concentration (pg/mL) ± SEM (one-way ANOVA; *asterisks above bars* indicate significance from control; *p < 0.05, **p < 0.01, ***p < 0.001, ****p < 0.0001)

To determine the signaling pathways regulating microglial cross communication with astrocytes, we treated microglia with the NF-κB inhibitor Bay 11-7082 prior to treatment and collection of MCM (Fig. 7). The ability of Bay 11-7082 to inhibit NF-κB-driven inflammatory gene expression was validated in BV2 microglia cells treated with LPS (1 μg/mL) for 24 h (Fig. 7a). Measured mRNA levels for *Nos2* were significantly reduced in LPS-treated BV2 cells pretreated with Bay 11-7082 compared to LPS or LPS + vehicle (DMSO). Treatment with Bay 11-7082 alone had no significant effect on *Nos2* expression. Pretreatment for 3 h with Bay 11-7082 also inhibited primary microglial expression of *Nos2* (Fig. 7b), *Tnf* (Fig. 7c), and *Caspase 1* (*Casp1*; Fig. 7d) compared to treatment with Mn alone or Mn + vehicle. Protein analysis via multi-plex and single-plex ELISA (Fig. 7e–j) showed a corresponding reduction in the release of TNF (Fig. 7g), IL-6 (Fig. 7h), CCL2 (Fig. 7i), and CCL5 (Fig. 7j). Astrocytes exposed to MCM from microglia treated with Bay 11-7082 had significantly reduced expression of *Tnf* (Fig. 7k) and *Ccl5* (Fig. 7o) as compared to treatment with MCM from Mn-treated microglia without Bay 11-7082. Similar trends toward decreased expression of *Il-1β* (Fig. 7l), *Il-6* (Fig. 7m), and *Ccl2* (Fig. 7n) were also noted in astrocytes treated with MCM from Bay 11-7082-treated microglia.

### Release of TNF by Mn-treated microglia plays an important role in inflammatory activation of astrocytes

**Fig. 8:**
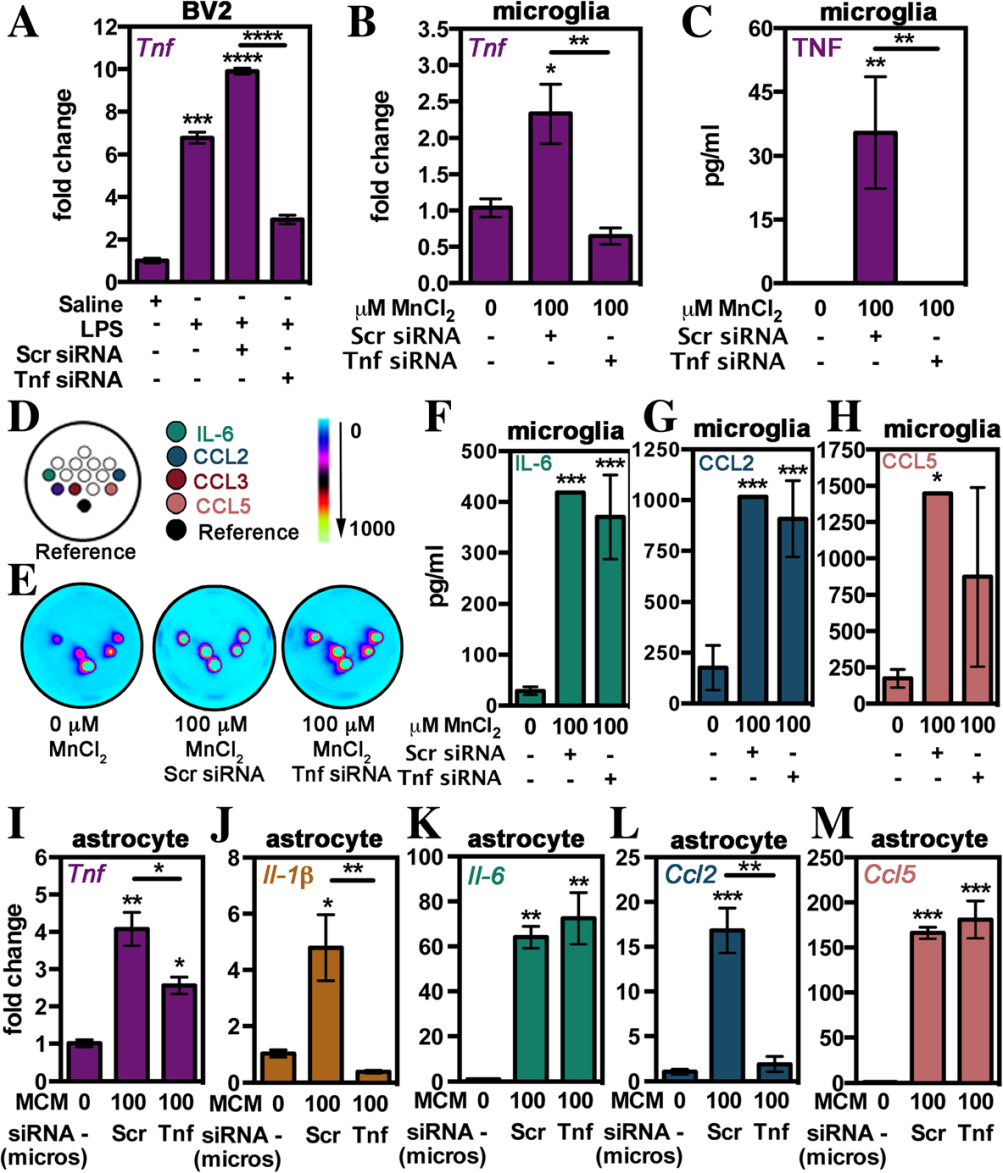
Release of TNF by Mn-activated microglia partly regulates inflammatory microglia-astrocyte crosstalk. *Tnf* knockdown in microglia was achieved through use of siRNA treatment 48 h prior to MCM experiments. **a** Successful knockdown of *Tnf* was assessed in BV2 microglial cells treated with lipopolysaccharide (1 μg/mL) for 24 h after 48 pretreatment with scrambled (Scr) siRNA or *Tnf* siRNA. **b** Knockdown of *Tnf* in primary microglia was assessed via qPCR. **c** TNF levels in MCM media prior to placement on astrocytes was assessed via single-plex TNF ELISA. **d** Representative image from the Quansys mouse 14-plex array indicating location of noted chemokines and cytokines. **e** Representative heat maps of Quansys multi-plex ELISA results of media from 0 or 100 μM MnCl_2_-treated microglia in combination with Scr siRNA or *Tnf* siRNA with dot locations correlating to **d**. Intensity is represented by no expression (*blue*) to increasing expression (*yellow*). Levels of inflammatory gene expression in astrocytes treated with MCM of microglia treated with Scr siRNA or *Tnf* siRNA were determined via qPCR for *Tnf* (**i**), *Il-1β* (**j**), *Il-6* (**k**), *Ccl2* (**l**), and *Ccl5* (**m**). Expression data is represented by mRNA fold change ± SEM while ELISA data is represented as mean concentration (pg/mL) ± SEM (one-way ANOVA; *asterisks above bars* indicate significance from control; *p < 0.05, **p < 0.01, ***p < 0.001, and ****p < 0.0001)

TNF is considered a master inflammatory regulator in the CNS that can induce production of additional cytokines leading to gliosis, demyelination, inflammation, and immune reactivity. Increased expression and release of TNF by microglia often occurs early in neurodegenerative diseases such as PD and human immunodeficiency virus-induced dementia and appears to be important for subsequent glial reactivity [44]. To investigate if TNF release by microglia is important for neuroinflammatory activation of astrocytes during exposure to Mn, we knocked down *Tnf* expression in microglia using a 48-h pretreatment with siRNA (Fig. 8). Knockdown was validated via qPCR in BV2 mouse microglial cells treated with with 1 μg/mL LPS. Measured mRNA levels for *Tnf* were significantly reduced after 24-h treatment in BV2 cells transfected with *Tnf* siRNA but not in cells transfected with control scrambled siRNA oligonucleotides (Fig. 8a). Furthermore, *Tnf* siRNA reduced *Tnf* expression (Fig. 8b) and TNF production (Fig. 8c) in purified primary microglia treated with 100 μM MnCl_2_. Analysis of MCM from siRNA-treated microglia (Fig. 8d–h) revealed that TNF levels were selectively reduced, whereas levels of IL-6 (Fig. 8f), CCL2 (Fig. 8g), and CCL5 (Fig. 8h) were unaffected. Analysis of inflammatory gene expression in astrocytes following treatment with MCM from microglia treated with *Tnf* siRNA revealed significant reduction in expression of *Tnf* (Fig. 8i), *Il-1β* (Fig. 8j), and *Ccl2* (Fig. 8l)*.* MCM-induced expression of *Il-6* (Fig. 8k), *Ccl5* (Fig. 8m), or *Nos2* (data not shown) in astrocytes was unaffected by *Tnf* knockdown in microglia.

## Discussion

Activation of microglia is central to neuroinflammation and occurs early in the pathogenesis of neurodegenerative diseases such as PD [23, 24] and Mn neurotoxicity [45]. Although microgliosis is well described in both humans and animals exposed to Mn [5, 46], mechanisms by which microglia contribute to the etiology and progression of Mn neurotoxicity are poorly defined. In this study, we sought to determine the role of microglia in modulating neuroinflammatory responses in Mn neurotoxicity by investigating the activated phenotype adopted by microglia exposed to Mn and the subsequent effect that Mn-induced microglia activation had on Mn-exposed astrocytes. Using immunopurified cultures of primary microglia and astrocytes, our results demonstrate that microglia directly accumulate Mn and develop a mixed inflammatory phenotype characterized by release of IL-6, TNF, CCL2, and CCL5. Furthermore, we show that products from Mn-activated microglia are essential for neuroinflammatory activation of Mn-exposed astrocytes at concentrations up to 100 μM Mn and that NF-κB-dependent release of TNF from microglia is a key signaling event in microglia regulating these glial-glial interactions.

Researching microglial responses in vitro has been hindered by the inability to obtain a high yield of purified cells due to low percentage of microglia in the healthy CNS [13], lack of proliferation in culture [47], and loss of cells when extracted from mixed glial cultures [30]. These inherent difficulties have resulted in many studies relying on immortalized microglial cell lines, such as BV2 and N9 cells, to study microglial responses. However, although these models have value in interrogating cellular signaling mechanisms, cell lines have been proven to require higher doses of toxicants to reach activation compared to primary cells, have more limited cytokine and chemokine profiles, and often do not undergo the prototypical morphological changes in response to activation [48, 49]. To avoid these limitations in immortalized cell lines, we used a described immunopurification method known to generate high numbers of purified microglia [30]. Evaluation of microglia purity by flow cytometry or immunofluorescence was 97%, similar to the purity of microglia cultures reported for shaking or Percoll extraction methods [50], but with a much higher yield of cells. This method also allowed for use of microglia-depleted astrocyte cultures with purities calculated above 90% using immunofluorescence. Determination of the percent purity of astrocyte cultures following removal of microglia using the markers GFAP or GLAST via immunofluorescence or flow cytometry indicated purities of 70–80%, similar to other published studies [50]. However, astrocytes are a heterogeneous cell population and do not uniformly express any given marker, thus requiring multiple methods to validate purity [50–53].

Most studies of Mn in microglia have relied on immortalized microglia cells to evaluate inflammatory responses to exposure, citing that microglia require either large doses of Mn above 500 μM or concurrent treatment with LPS or cytokines to elicit inflammatory activation [6, 8, 54]. This is in contrast to studies using primary rat microglia where lower doses of Mn below 100 μM resulted in upregulation of *Tnf* and *Il-1β* [55] and production of reactive oxygen species without co-activators [56]. Using primary murine microglia, we also found that Mn at doses at or below 100 μM was sufficient to induce the expression of inflammatory genes in microglia, including *Nos2*, *Tnf*, *Il-6*, *Il-1β*, and *caspase 1*. Additionally, we report that primary microglia directly uptake Mn, as measured by CFMEA in live cells and by analytical determination of Mn remaining in microglia-conditioned media by ICP-MS. Studies in astrocytes report that both the transferrin receptor and the divalent metal transporter-1 act as putative receptors for Mn uptake in these cells [2, 57] and microglia are known to express both of these transport proteins [58]. However, additional studies are required to determine if these receptors are also involved in Mn uptake in microglia and if uptake is required for microglia activation.

As a myeloid lineage cell, microglia can polarize into a M1 (inflammatory) or M2 (anti-inflammatory) phenotype upon immune activation [15, 18]. The putative microglia phenotype that is predominately adopted with any given stimulus has shown to play an important role in determining the effect of the neuroinflammatory response on neuronal survival and astrocyte activation and proliferation [59–61]. Polarization can also dictate cross communication between astrocytes and microglia as highlighted in glial crosstalk studies for contusive brain injury. In those models, M1-stimulated microglia activate astrocytes that in turn release factors that further promote inflammatory activation and release of TNF in microglia/macrophages [61]. To further characterize microglial responses in Mn toxicity, we therefore examined gene expression of protypical M1 versus M2 genes in Mn-stimulated microglia. These gene studies revealed a mixed M1/M2 phenotype with no clear indication of a predominant phenotype at the time point studied. Other models examining polarization have also seen similar results, whereby M1 and M2 populations often co-exist and show overlapping effects [14, 42] or vary over time [62]. Further examination of Mn-stimulated polarization throughout the time course of exposure or the use of fluorescence-activated sorting to precisely count the percentage of each phenotype could be useful in more precisely determining the degree of polarization at particular concentrations of Mn [63].

After establishing that Mn activated microglia into a mixed inflammatory phenotype, we wanted to determine how Mn-activated microglia could influence astrocyte responses in the presence of Mn. In both the conditioned media and co-culture model, astrocytes required microglia exposed to Mn to fully mount an inflammatory response in the presence of Mn at the doses utilized (at a 1:2 ratio of microglia to astrocytes). In the absence of microglia, Mn treatment alone was insufficient to increase expression of inflammatory genes in astrocytes, except for *Nos2*, even at the highest dose examined (100 μM). These results may help to explain why previous studies in our laboratory and others that examined astrocyte inflammatory responses to Mn reported that cytokines, presumably from microglia, were required to activate astrocytes in the presence of low micromolar concentrations of Mn [7, 64]. The results of the present study also corroborate earlier findings that microglia were required for Mn-induced neuronal death in mixed glial-neuronal cultures [55]. Because our study only looked at relatively low doses of Mn, the requirement for the presence of microglia to activate astrocytes may not be same at higher doses of Mn, given that other studies do not report toxic changes in astrocytes unless Mn concentrations range above 500 μM [65].

The degree of inflammatory gene induction in astrocytes differed between the two culture systems, with conditioned media causing almost twofold greater induction in inflammatory gene expression than co-culturing astrocytes with microglia. This is most likely explained by the presence of astrocytes in the co-culture system. Astrocytes are known to sequester the majority of Mn in the brain [66] and thus likely influenced the amount of Mn available for microglia uptake. Additionally, studies focused on the effects of astrocytes on microglia reveal a trend whereby the presence of astrocytes reduces microglia release of reactive oxygen/nitrogen species [67, 68] and TNF [61] while promoting microglial release of anti-inflammatory factors such as IL-10 [63]. Thus, in the co-culture system but not in the conditioned-media system, the presence of astrocytes could influence the degree of neuroinflammatory gene expression in microglia and thereby modulate microglial responses to Mn. This makes the co-culture system a more physiological model than the MCM experiments; however, MCM experiments were necessary to allow assessment of cellular origin of released factors and selective cellular inhibition.

Microglia are considered essential for the development of an activated phenotype in astrocytes [44] because they release soluble factors that stimulate signaling pathways in astrocytes [69] leading to production of neurotoxic inflammatory mediators [24]. Studies examining microglial factors involved in modulating astrocyte responses have consistently identified the cytokines TNF, IL-1β, and IL-6 as critical to inflammation, because inhibiting or removing these factors greatly reduces astrocyte activation [16, 24, 69, 70]. Similar to other neuroinflammatory models, we detected increased production of IL-6 and TNF in MCM from Mn-treated microglia. In addition, multi-plex ELISA assays also identified increased production of the chemokines CCL2 and CCL5. Chemokines in the CNS are utilized to recruit immune cells, as well as microglia and astrocytes, to sites of injury [71] and are implicated as important factors in priming glial cells toward a more inflammatory phenotype [59, 72, 73]. However, there is little data on the impact of microglia-derived CCL2/CCL5 on astrocyte responses or the role of these chemokines in Mn neurotoxicity.

The NF-κB pathway is a primary regulator of cytokine and chemokine production [22] and has been shown to be vital in the inflammatory responses of both astrocytes [7, 74] and microglia [8] in response to Mn. Several signaling pathways including MAPK [8], p38 [75], and activated protein 1 (AP-1) have also been implicated in Mn-induced microglial activation but do not appear to be vital to microglia-astrocyte communication in response to Mn. Inhibition of microglial NF-κB signaling using the IKK complex inhibitor Bay 11-7082 resulted in significant reduction in microglia-induced astrocyte activation in our model. Microglial factors attenuated by NF-κB inhibition included IL-6, CCL2, CCL5, and TNF, but when TNF levels were reduced via RNAi, there was only partial reduction in astrocyte inflammatory gene expression. This indicates that additional signaling factors are likely involved in cross-communication leading to activation of astrocytes. TNF was required for induction of *Tnf*, *Il-1β*, and *Ccl2* in astrocytes but had no effect on *Il-6* or *Ccl5* expression. However, the level of *Il-6* and *Ccl5* mRNA expression was reduced with NF-κB inhibition when these factors were reduced in MCM. These results indicate that TNF is a major regulator of multiple inflammatory genes in microglia-to-astrocyte crosstalk, whereas IL-6 and CCL5 appear to regulate their own signaling and production across both glial cell types during Mn exposure.

## Conclusions

Manganese neurotoxicity results in an incurable and progressive movement disorder that currently lacks any definitive treatment options [76]. Neuroinflammatory activation of glia is thought to play a role in the progression of this disease and in promoting neurological injury from both acute and chronic exposure. Because glial responses to inflammatory cues can both protect and damage neurons depending on the degree of activation and the severity of the toxic exposure, examining the pathways and signaling events regulating crosstalk between glia will be critical to developing better modalities to treat neuroinflammation. With the paucity of information regarding the effects of microglia on astrocyte responses during Mn neurotoxicity, we provide evidence that microglia-astrocyte crosstalk through NF-κB signaling plays an essential role in inflammatory responses to Mn exposure. Continued research exploring how glial communication affects neuronal injury in this model will help our understanding of how glial-glial interactions regulate neuroinflammatory mechanisms in Mn neurotoxicity and during other neurotoxic insults.

## Abbreviations

Casp1: Caspase 1
CCL2: C-C motif chemokine ligand 2
CCL3: C-C motif chemokine ligand 3
CCL5: C-C motif chemokine ligand 5
CD11b: C3 complement receptor
ELISA: Enzyme-linked immunosorbent assay
GFAP: Glial fibrillary acidic protein
GLAST: Glutamate transporter
IBA-1: Ionized calcium binding adaptor molecule 1
IL-1β: Interleukin-1 beta
IL-6: Interleukin-6
Mn: Manganese
NF-κB: Nuclear factor kappa B
NOS2: Nitric oxide synthase 2
TNF: Tumor necrosis factor alpha
